# Supplementary material for: Programmable phase-change metasurfaces on waveguides for multimode photonic convolutional neural network
Source: Nat Commun. 2021 Jan 4;12:96. doi: 10.1038/s41467-020-20365-z (PMC7782756; doi:10.1038/s41467-020-20365-z)
Supplement: Supplementary file 1 — Supplementary Information [file 41467_2020_20365_MOESM1_ESM.pdf]

Supplementary Information for

Programmable Phase-change Metasurfaces on Waveguides for Multimode Photonic Convolutional Neural Network

Changming Wu[1], Heshan Yu[2], Seokhyeong Lee[1], Ruoming Peng[1], Ichiro Takeuchi[2], and Mo Li[1,3]

[1]*Department of Electrical and Computer Engineering, University of Washington, Seattle, WA 98195, USA*

[2]*Department of Materials Science and Engineering, University of Maryland, College Park, MD 20742, USA*

[3]*Department of Physics, University of Washington, Seattle, WA 98195, USA*

Supplementary Note 1: Phase-change material characterization

The 30 nm thick $Ge_2Sb_2Te_5$ (GST) thin film used in this work is sputtered on a silicon nitride-on-insulator substrate at room temperature. A 10 nm thick SiO_2 layer is then sputtered to cover the GST film to prevent oxidation and degradation during the fabrication and measurement processes. The refractive index n, as well as the extinction coefficient κ of the GST measured by a spectroscopic ellipsometer, is shown in Supplementary Figure 1.

Experimentally, GST can be programmed to an intermediate phase between a fully amorphous and a fully crystalline phase. The effective refractive index n and extinction coefficient κ for mixed phases (partially crystallized and partially amorphous) can be estimated from effective permittivity approximation, calculated with the effective-medium $\varepsilon_{\mathrm{eff}}$ theory[1,2]:

$$\frac{\varepsilon_{\mathrm{eff}}(p)-1}{\varepsilon_{\mathrm{eff}}(p)+2}=p\times\frac{\varepsilon_{\mathrm{c}}-1}{\varepsilon_{\mathrm{c}}+2}+(1-p)\times\frac{\varepsilon_{\mathrm{a}}-1}{\varepsilon_{\mathrm{a}}+2},$$

where ε_c, ε_a are the complex permittivities measured using ellipsometry spectroscopy for cGST and aGST phases respectively and can be obtained from $\sqrt{\varepsilon}=n+i\kappa$, p is the percentage of crystallization, so p=100% corresponds to the fully cGST phase while 0% corresponds to the fully aGST phase.

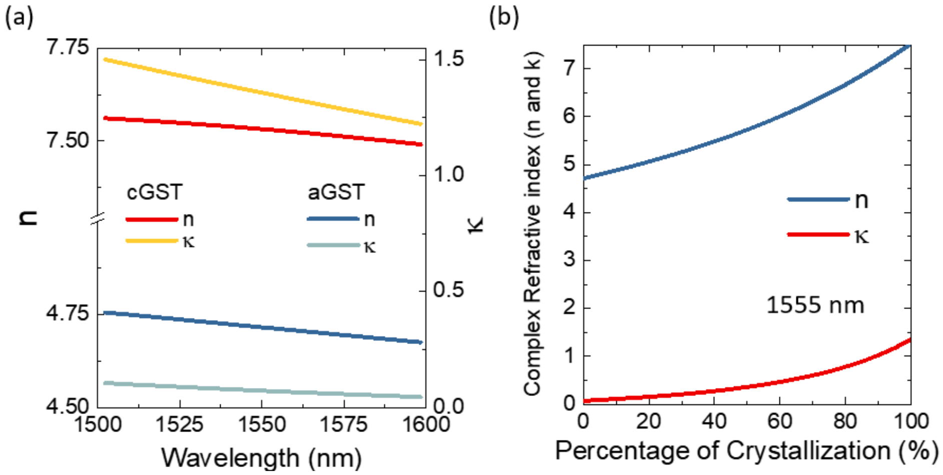

Supplementary Figure 1 (a) Refractive index n and extinction coefficient κ of the crystalline and amorphous phases of $Ge_2Sb_2Te_5$ measured with ellipsometry. (b) The change of n and κ with the percentage of crystallization p.

Supplementary Note 2: Phase-change metasurface mode converter (PMMC) design

The phase-change material based phase-gradient metasurface utilizes the consecutive scattering of the waveguide mode by the GST nano-antenna array. To build a well-defined phase gradient $d\Phi/dx$, the phase response of a single GST nano-antenna is simulated first[4]. The inset of Fig. 1c in the main text shows the crosssection of the geometry used in the simulation, in which a single GST nano-antenna with various width is placed on a 1.8 μm wide, 330 nm thick Si_3N_4 multimode waveguide, with a 400 nm offset from the central axis of the waveguide. The waveguide and the GST nano-antenna are conformally covered with a layer of 218 nm thick Al_2O_3 layer as a protection layer. A fundamental waveguide TE_0 mode is launched into the waveguide. The field distribution right after the TE_0 mode pass through the nano-antenna is recorded. To obtain the scattered phase precisely, we also perform another simulation with similar device geometry but without a GST nano-antenna as a reference, which is subtracted from the first simulation to calculate the scattered field by the nano-antenna only. In this way, the phase and amplitude information of the scattered field by the nano-antenna can both be determined. In the simulation, we sweep the width of the nano-antenna as well as the phase of the GST (both aGST and cGST). Since cGST has a higher refractive index near 1550 nm, the field scattered by cGST nano-antenna is much stronger compared to the field scattered by aGST nano-antenna. The phase response also shows a much stronger dependence on nano-antenna width when the GST is in the crystalline phase compare to when GST is in the amorphous phase. Thus, consider both the phase and the amplitude response together, a phase-gradient metasurface mode converter designed for the cGST nano-antenna won't be effective when the GST is in the aGST phase, other than causing a small perturbation to the mode.

To construct the metasurface, we arrange a set of cGST nano-antennas with varying widths into an array with a subwavelength spacing of *dx* between adjacent ones, and set the phase response between two adjacent nano-antennas to be $d\Phi$. Thus a constant phase gradient of $d\Phi/dx$ along the gradient metasurface is created. Since we only monitor the phase and amplitude responses of a single isolated nano-antenna as described above, when we arrange many nano-antennas into an array, the near-field inter-nano-antenna coupling between adjacent nano-antennas introduces a small additional perturbation, causing a variation of the phase gradient. To optimize the mode converter performance, we perform several rounds of optimization of the metasurface. The parameters to be optimized include the thickness of the Al_2O_3 encapsulation layer, the lengths and the widths of the GST nano-antennas. The parameters after optimization are listed in Supplementary Table 1.

Central wavelength (nm)	1550
Dimensions of the waveguide cross-section (µm × µm)	1.8 × 0.33
Al_2O_3 layer thickness (nm)	218
Numbers of antennas	25
Antenna length/thickness/spacing (nm)	172/ 30/ 400
Phase incremental (degrees)	2.5
Antenna offset from waveguide central axis (nm)	400
Antenna lengths (nm)	510, 480, 447, 425, 398, 380, 358, 341, 323, 308, 290, 278, 261, 248, 233, 220, 205, 192, 177, 164, 149, 135, 119, 105, 84

Supplementary Table 1 Optimized parameters of the programmable phase-change material based metasurface in the mode converter.

Supplementary Note 3: Simulation on the performance of the PMMC

One feature for this metasurface mode converter is that it supports asymmetric optical power transmission when light travels along with two opposite directions[4]. Here we define the "forward direction" as the propagation direction following the tapering of the width of the nano-antenna while the "backward direction" is the opposite direction. The mode converter is designed to work under the crystalline phase GST. As shown in Supplementary Figure 2, when GST is in the aGST phase, the incident TE_0 mode propagates in both forward and backward directions without being converted, so it is the dominant mode at the output in both directions. The slight difference at the output is due to the broken mirror symmetry caused by the GST array. When the GST in the cGST phase, the incident TE_0 mode propagating in the forward directions will be converted to TE_1 mode while the mode propagating in the backward directions will be

scattered out,so the transmittance will be reduced. Besides, due to the high absorption of the cGST (high κ), the total insertion loss is higher compared to the insertion loss of the aGST phase. Recently, new phase-change materials combine broadband transparency with large optical contrast have been reported[5,6], which can help further reduce the insertion loss.

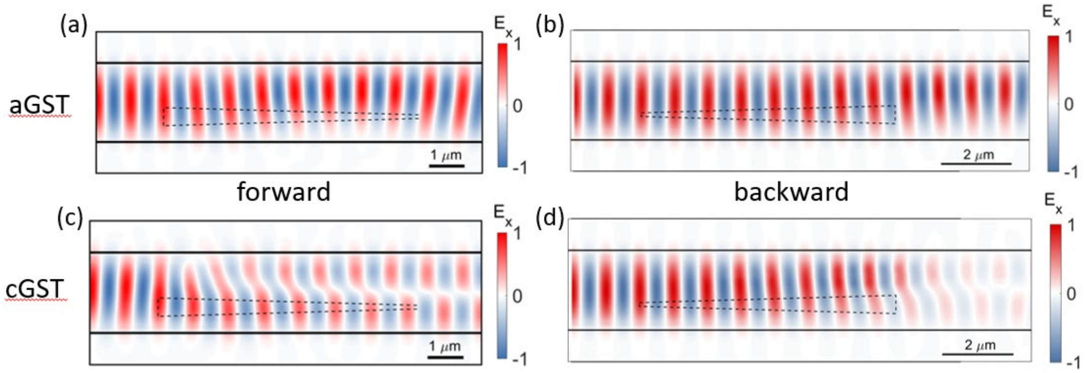

Supplementary Figure 2 Simulation results showing the incident fundamental TE_0 mode evolving when propagating in the PMMC. **(a, b)** When GST is in the aGST phase, the mode conversion does not happen both in the forward propagation direction (a) and the backward direction (b). **(c, d)** When GST is in the cGST phase, the incident mode will gradually be converted in the TE_1 mode in the forward propagation direction (c) and scattered out of the waveguide as surface waves in the backward direction (d). The black dashed lines outline the profile of the nano-antenna array.

One of the advantages of using the GST metasurface is that the GST can be controlled between a fully amorphous and a fully crystalline phase using optical pulses, thus bring about the programmability that is needed for reconfigurable photonics and optical computing. We simulate the performance of the PMMC in Supplementary Figure 3 assuming the phase of the GST metasurface can be tuned continuously and uniformly. The optical properties, the refractive index n and the extinction coefficient κ, are taken from the Supplementary Figure 1b. Supplementary Figure 3a to 3c show the 2D plot of the normalized transmission T, TE_1 mode purity β_{TE1}, and mode contrast Γ, as defined in the main text and respectively, when the wavelength and the crystallization percentage are swept. The transmission spectra at various crystalline percentages are normalized by the transmission spectrum at the amorphous phase. As shown in the simulation, the PMMC performs well over broadband ranging from 1500 nm to 1600 nm. When the GST metasurface is in the cGST phase, though the transmission of the TE_1 mode is low due to cGST's absorption, the TE_1 mode purity is >95% so most of the transmitted power is carried by the TE_1 mode. In contrast, when the GST metasurface is in aGST phase, the TE_0 mode purity is as high as 87%, so most of the transmitted power is carried by the TE_0 mode. The detailed cross-sectional line cut of the 2D plot is also shown in Supplementary Figure 3d, e. Supplementary Figure 3f shows that changing the GST from fully crystalline to fully amorphous step by step, the mode contrast is varied stepwise from -1.0 to +0.75.

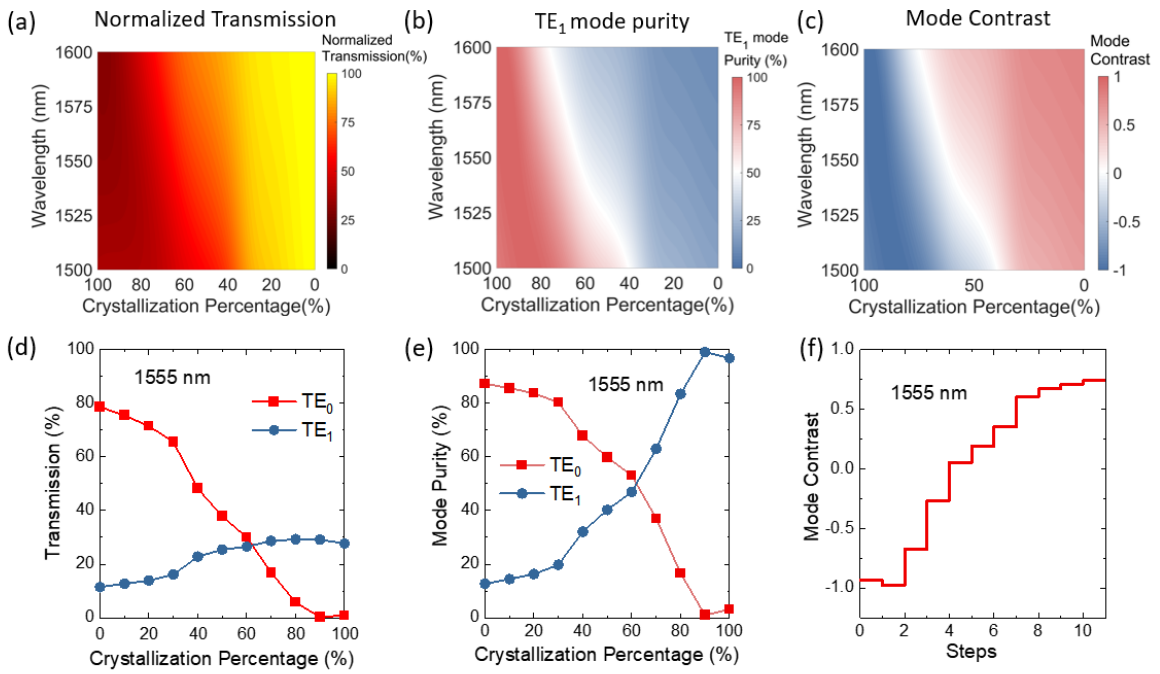

Supplementary Figure 3 Simulation results of the performance of the mode converter. **(a)-(c)** 2D plots of the normalized transmission (a), the TE_1 mode purity (b) and the mode contrast (c), when both the optical wavelength and the crystallization percentage are scanned. **(d)-(e)** Cross-sectional plot of the transmission (d) and the mode purity (e) as a function of the crystallization percentage (0% corresponds to cGST and 100% corresponds to aGST). **(f)** Simulation of the mode contrast varies when the GST's phase is changed from cGST to aGST step by step. Step 0 corresponds to the fully crystalline phase. In each step, the crystallization percentage drops by 10%. The crystallization percentage drops to 0 (fully amorphous phase) at step 10.

We also performed FDTD simulations on the performance of the antenna array when sweeping the array period or the length of the antenna and plotted the output TE_1 mode purity at the 1570 nm. When we swept the array period or the antenna length, the other parameters are kept the same as we used in the main text and the detailed parameters can be found in Supplementary Table 1.

Supplementary Figure 4a shows the TE_1 mode purity for both cGST and aGST as a function of the array period. The star marks the purity at the period we used in the main text(0.4 μm). The inset denotes the definition of the array period, which is the distance between two adjacent antennas. Changing the array period will change both the phase gradient and the coupling strength between the TE_0 and TE_1 modes. As shown in Supplementary Figure 4a, for aGST, the TE_1 mode purity will gradually decrease when the array period increases from 0.2 μm to 0.9 μm, while for cGST, β_{TE1} will have its maximum at the antenna period of 0.4 μm. When the array period larger than 0.7 μm, the period is comparable to the effective wavelength of the mode, the phase gradient is negligible. The incident light is scattered by several independent antennas, thus β_{TE1} is a constant for both phases. Therefore, we choose a period of 0.4 μm to optimize the contrast of β_{TE1} when GST is switched between the two phases.

Supplementary Figure 4b shows the TE_1 mode purity for both the cGST and aGST phases as a function of the antenna length. The star marks the purity at the length we used in the manuscript (0.172 μm). The inset denotes the definition of the antenna length. Changing the antenna length will change the coupling strength between the TE_0 and TE_1 modes but only slightly change the phase gradient. If the antenna length is too small, the coupling is too weak; thus β_{TE1} is small for both phases of GST. The β_{TE1} increases with the increasing antenna length for aGST. For cGST, as the length of antenna increases, the β_{TE1} first increases rapidly until it reaches its maximum at the antenna length around 0.15 μm and then gradually decreases.

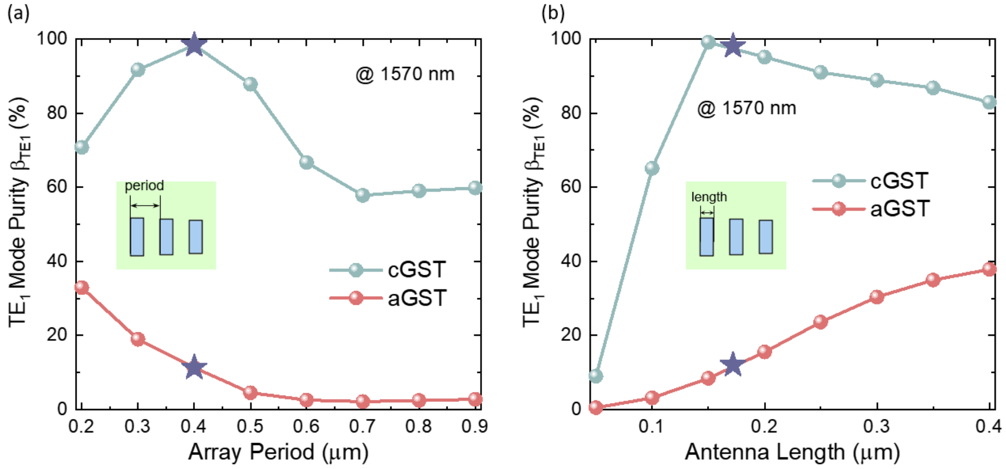

Supplementary Figure 4 (a) Calculated TE_1 mode purity for both cGST and aGST phases, as a function of the array period. The star marks the period we used in the experiment (0.4 μm). The inset denotes the definition of the array period, which is the distance between two adjacent nano-antennas. (d) Calculated TE_1 mode purity for both cGST and aGST phases, as a function of the antenna length. The star marks the length used (0.172 μm) in the experiment. The inset denotes the definition of the antenna length.

We also performed FDTD simulations and plotted the TE_1 mode purity as well as the mode contrast of two geometries (nano-antenna array and tapered patch) in Supplementary Figure 5.

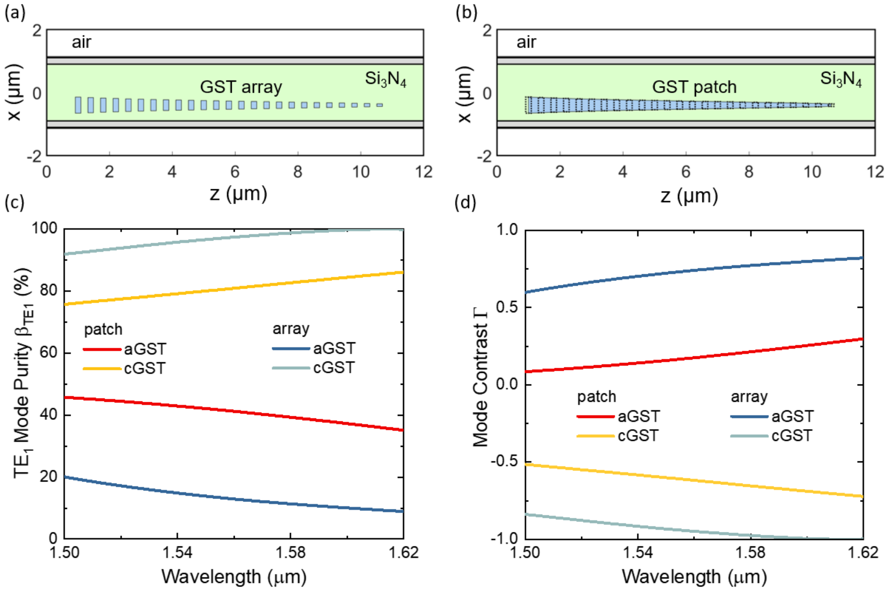

Supplementary Figure 5 (a)-(b): Top view (*x-z* cross-sectional plane) of the two geometries (GST antenna array and tapered patch). The green, blue, grey, white areas denote the Si_3N_4 waveguide, GST array, Al_2O_3 capping layer, ambient air, respectively. The dashed line in (b) outlines the nano-antenna array for comparison. **(c)-(d)**: The TE_1 mode purity (c) and mode contrast (d) as a function of the wavelength for both geometries.

Supplementary Figure 5a and 5b show the top view (*x-z* cross-sectional plane) of the two geometries (GST antenna array and tapered patch). The green, blue, grey, white areas denote the Si_3N_4 waveguide, GST array, Al_2O_3 capping layer, ambient air, respectively. The geometry of the nano-antenna array is the same as the device we have shown in the manuscript. The dashed line rectangles in Supplementary Figure 5b indicates the outline of the nano-antennas in Supplementary Figure 5a for comparison. The definition of the TE_1 mode purity β_{TE1} and the mode contrast Γ is the same as in the manuscript, $\beta_{\mathrm{TE0(TE1)}} = P_{\mathrm{TE0(TE1)}} / \left(P_{\mathrm{TE0}} + P_{\mathrm{TE1}}\right)$, $\Gamma = \beta_{\mathrm{TE0}} - \beta_{\mathrm{TE1}}$, where P_{TE0} (P_{TE1}) is the power in the TE_0 (TE_1) mode.

We compare the TE_1 mode purity for both cGST and aGST phases in two geometries, as shown in Supplementary Figure 5c. For the nano-antenna array geometry, the TE_1 mode purity changes from 92-99.9% in the whole spectrum range (from 1.50 μm to 1.62 μm) to 9-20% when the GST is switched from cGST to aGST phase. In comparison, the continuous tapered GST geometry has a much lower change, from 76-86% to 35-46%. This directly leads to a difference in mode contrast Γ changes, as plotted in panel Supplementary Figure 5d. For example, at 1.555 μm, Γ can be tuned from -0.93 to 0.72 in the antenna array geometry (experimental value: -0.73 to 0.67), while only from -0.61 to 0.17 in the single patch geometry. The large tunable change of Γ is critical for the use of it to represent the kernel element values in MVM computing, in terms of both precisions (up to 6 bits) and reliability.

The number N of the antenna in the array used in our device is optimized to achieve high TE_1 mode purity β_{TE1}. We calculate the TE_1 mode purity as a function of the antennas numbers in the array when the

GST is in the cGST phase, as shown in Supplementary Figure 6. As shown in the inset of the figure, the number of antennas is changed while keeping the phase gradient a constant. The results show that β_{TE1} is non-monotonically dependent on the number of antennas with a peak at N=23. Beyond that, β_{TE1} actually decreases with N, because of the increasing insertion loss. In our experiment, considering the fabrication deviation, we used N=25.

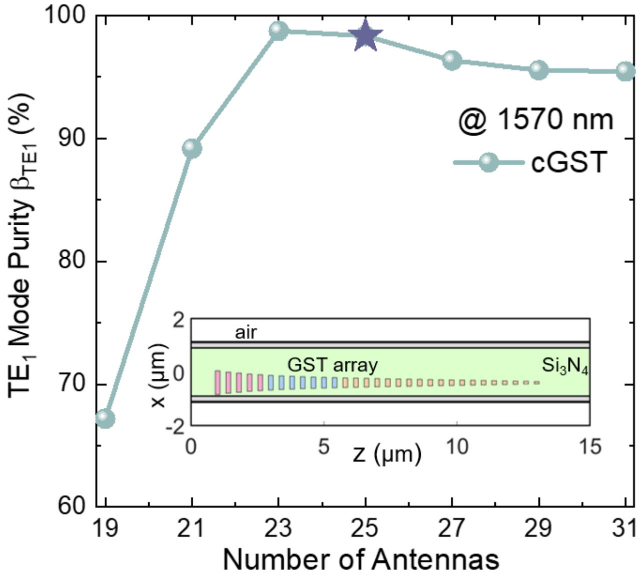

Supplementary Figure 6 The TE_1 mode purity as a function of the number of antennas. The number of antennas changes while keeping the phase gradient as a constant. The inset is the geometry of 19 (yellow), 26 (yellow+blue), 31 (yellow+blue+red) nano-antennas, respectively. The star symbol marks the number of nano-antennas (25) used in the experiment.

Supplementary Note 4: Device fabrication and fabrication error characterization

To fabricate the PCMM, we deposited a layer of 30 nm thick $Ge_2Sb_2Te_5$ with a layer of 10 nm thick SiO_2 film on top by sputtering on the silicon nitride on insulator wafer (330 nm thick stoichiometric silicon nitride film deposited by LPCVD on an oxidized silicon wafer). The metasurface was patterned with electron beam lithography system (EBL) using resist ZEP 520A, and etched with an inductively coupled plasma etching (ICP) system using fluorine-based chemistry. Next, the photonic structures such as waveguides, mode selectors and grating couplers are patterned and etched using the EBL and dry etching processes. Afterward, the GST nano-antenna array was conformally covered with a 218 nm thick Al_2O_3 layer deposited with atomic layer deposition (ALD) method followed by a standard lift-off process to complete the fabrication. After fabrication, the substrate was baked at 180 °C on a hotplate for 10 minutes to convert the GST into the fully cGST phase.

The deviation between the geometry of the designed and the fabricated nano-antennas is characterized by the SEM images taken before the Al_2O_3 encapsulation process and are listed in Supplementary Figure

10e. The fabrication process is optimized to make the nano-antennas in the intermediate part of the array as accurate as possible, with <5% deviation from design. The overall errors in the widths of fabricated nano-antennas are controlled within ~15% except for the shortest nano-antennas. Despite the fabrication errors, the devices have excellent programmable mode conversion performances, demonstrating the robustness of the design against fabrication errors[4].

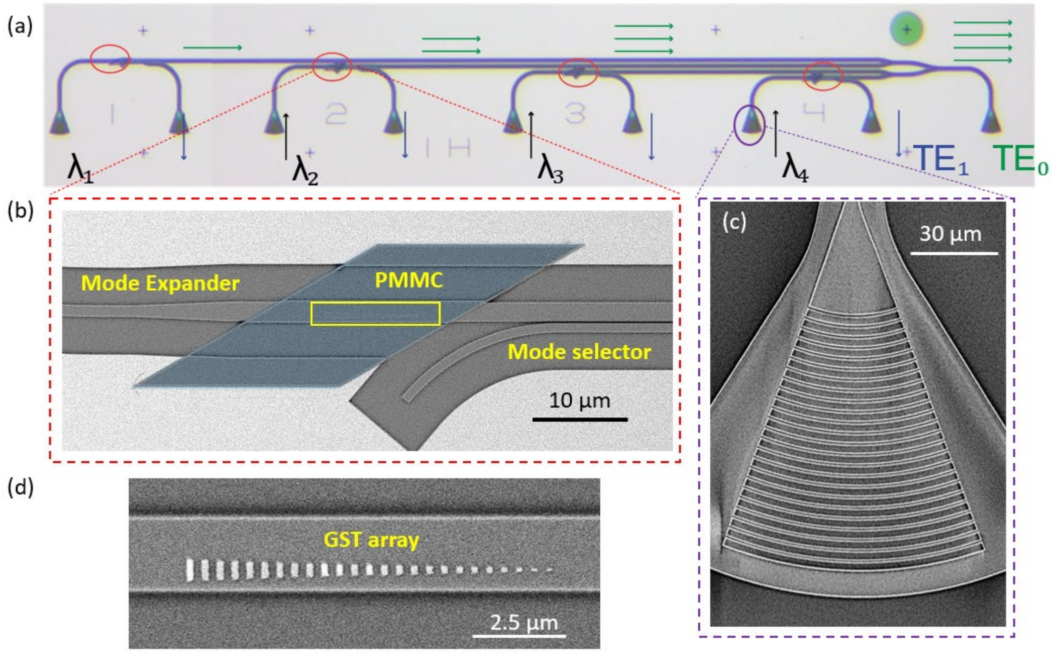

Supplementary Figure 7 (a) The blow-up optical image of the same device as shown in Fig. 3b in the main text. **(b)** The enlarged mode converter region, including a mode expander, a PMMC, and a mode selector. The blue shaded area is the Al_2O_3 capping layer. **(c)** SEM image of the grating coupler. **(d)** SEM image of the GST array before Al_2O_3 encapsulation.

Supplementary Figure 7 shows a blow-up image of the device, as in Fig. 3b of the main text, with more details. The whole device consists of four programmable mode converters. As shown in Supplementary Figure 7b, a mode expander is connected to the mode converter and followed with a mode selector, and the mode converter is encapsulated by a layer of Al_2O_3 (the blue shaded area). The incident light is firstly coupled into the single-mode waveguides through grating couplers (See Supplementary Figure 7c for the SEM image of the grating coupler). The mode expander is used to expand the incident TE_0 mode from the single-mode waveguide to the multimode waveguide. After the mode conversion, the light passes through the mode selector, which is an asymmetric directional coupler, to separate the TE_0 and TE_1 mode components at the output. The TE_1 mode components would couple out from separate ports and are summed up off-chip. The TE_0 mode components are summed on-chip using Y-junctions and coupled out of the chip.

Supplementary Note 5: Mode selector and mode expander design

Asymmetric directional couplers are designed to separate the TE_1 mode from the TE_0 mode in the multimode waveguide. As shown in Supplementary Figure 8, the mode selector consists of a single-mode coupling waveguide and a multimode bus waveguide. The widths of the coupling waveguide and the bus waveguide are 0.91 µm and 1.8 µm, respectively. The gap between the two waveguides is 150 nm and the coupling length is around 22 µm. The waveguide widths are designed to equalize the effective mode indices of the TE_0 mode in the coupling waveguide with the TE_1 mode in the bus waveguide so they can couple with each other. The effective mode index of the TE_0 mode in the bus waveguide, however, is very different from that of any modes in the coupling waveguide, so it will not couple to the coupling waveguide. As demonstrated in the simulation in Supplementary Figure 8a and b, the mode selector separates the TE_1 from the TE_0 modes in the bus waveguide to the coupling waveguide.

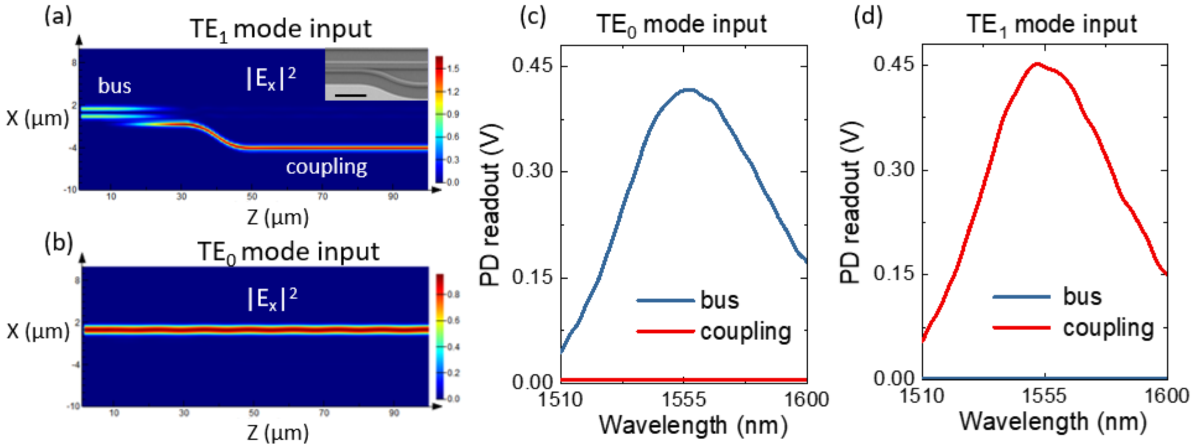

Supplementary Figure 8 (a) and (b): Simulation results of mode propagation through the mode selector. If the input in the bus waveguide is in the TE_1 mode, it will gradually couple to the coupling waveguide. If the input mode is the TE_0 mode, it will stay in the bus waveguide. Inset: SEM image of the mode selector. Scale bar: 10 µm. **(c)** and **(d)**: Experimental characterization of the mode selector.

To characterize the performance of the mode selector experimentally, we fabricate a pair of the mode selector connected with the multimode waveguide, the front one is used to input the modes and the back one is used to select the mode components. As demonstrated in Supplementary Figure 8c and d, if the TE_1 mode is input, it will output to the coupling waveguide; if the TE_0 mode is input, in contrast, it will stay in the bus waveguide. The performance of the mode selector is used as a reference when we measure the mode-conversion performance of our PMMC.

The mode expander is a 10 µm-long tapered waveguide connected with a 0.91 µm-wide single-mode waveguide and a 1.8 µm-wide multimode waveguide at the two ends respectively. The mode expander is

used to expand or squeeze the TE_0 mode while blocking the incident TE_1 mode from the multimode waveguide, as shown in Supplementary Figure 9.

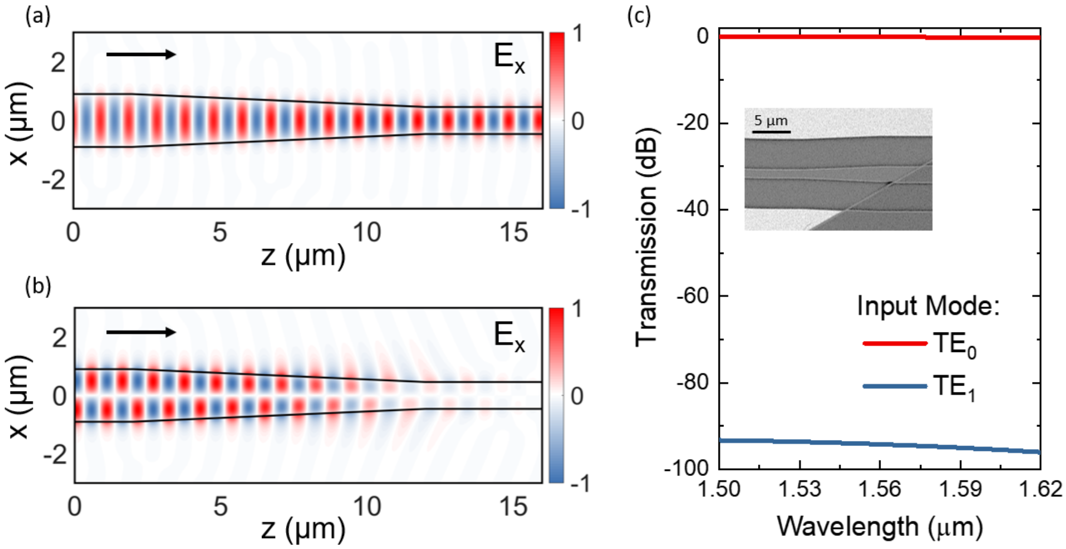

Supplementary Figure 9 Simulation results of mode propagation in the mode expander from a multimode waveguide to a single-mode waveguide. The wavelength is 1550 nm. The arrow indicates the propagation direction. **(a)** The input TE_0 mode will pass the mode expander with the expanded or squeezed mode profile with high efficiency. **(b)** TE_1 mode will experience a high loss. **(c)** The transmission spectrum in dB showing a high transmission for TE_0 mode (insertion loss < 0.2 dB over broadband) and a high loss for the TE_1 mode (insertion loss > 90 dB over broadband). The inset shows the SEM image for the mode expander.

Supplementary Note 6: Additional experimental data on the performance of the PMMC

Supplementary Figure 10a and b show when a TE_0 mode incidents into the PMMC, as the phase of the GST is gradually quenched from cGST to aGST, the TE_0 mode transmitted power at the output rises while the TE_1 mode transmitted power drops. Thus the PMMC can be programmed by controlling the phase of the GST metasurface.

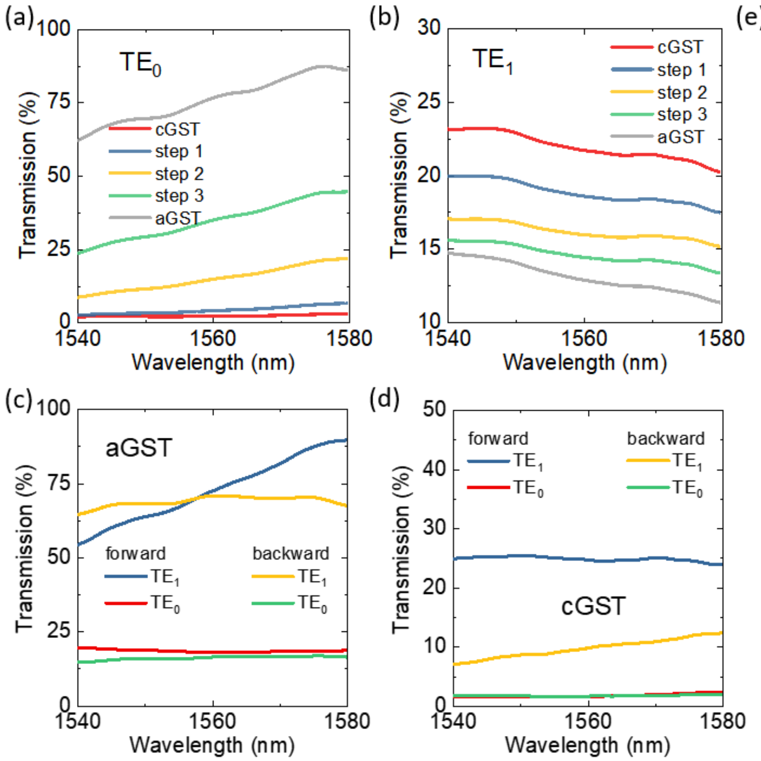

(e)

#	Design (μm)	Fabrication (μm)	%Diff
1	0.510	0.576	12.94
2	0.480	0.525	9.38
3	0.447	0.505	12.98
4	0.425	0.484	13.88
5	0.398	0.433	8.79
6	0.380	0.428	12.63
7	0.358	0.398	11.17
8	0.341	0.358	4.99
9	0.323	0.353	9.29
10	0.308	0.336	9.09
11	0.290	0.291	0.34
12	0.278	0.288	3.60
13	0.261	0.273	4.60
14	0.248	0.252	1.61
15	0.233	0.241	3.43
16	0.220	0.218	0.91
17	0.205	0.211	2.93
18	0.192	0.202	5.21
19	0.177	0.167	5.65
20	0.164	0.158	3.66
21	0.149	0.135	9.40
22	0.135	0.118	12.59
23	0.119	0.102	14.29
24	0.105	0.066	37.14
25	0.084	0.055	34.52

Supplementary Figure 10 (a)-(b) The TE_0 mode **(a)** and TE_1 mode **(b)** transmission coefficients when GST is gradually quenched from a fully amorphous phase to a fully crystalline phase. **(c)-(d)**: When GST is at aGST (c) and cGST (d) phases, the transmission coefficient of TE_0 and TE_1 modes, when the incident TE_0 mode is propagating in forward and backward directions. **(e)** The designed width, fabricated width, and their difference of each nano-antenna in the metasurface.

The performance of the PMMC strongly depends on the phase of the GST. To confirm this, Supplementary Figure 10c and d show the measured transmission spectra of the PMMC as the incident light is propagating along with the forward and the backward direction. When the GST is in the cGST phase, the device shows a strong asymmetric transmission behavior. The light is strongly scattered out when the light travels backward as described above in Supplementary Note 3, thus the transmitted power is suppressed. When GST is in the aGST phase, the PMMC is ineffective with only a slight asymmetric transmission spectrum can be measured.

To compare with the simulation results in Supplementary Note 3, Supplementary Figure 11 plots experimentally measured results of the normalized transmission, the TE_1 mode purity, and the mode contrast as a function of the wavelength and the phase of the GST. Since we quench the GST from cGST to aGST step by step using optical pulses, the crystallization percentage gradually decreases. The step “0” corresponds to the cGST phase and step “1” corresponds to the aGST phase. Compare with the simulation results in Supplementary Figure 3, the *x*-axis has a distortion. This is because in the simulation, we assume the phase of the GST array varies continuously and uniformly. However, in a real experiment, the

crystallization percentage cannot change continuously, and the phase of each nano-antenna is not uniform either. Nevertheless, our experimental results agree well with the simulation.

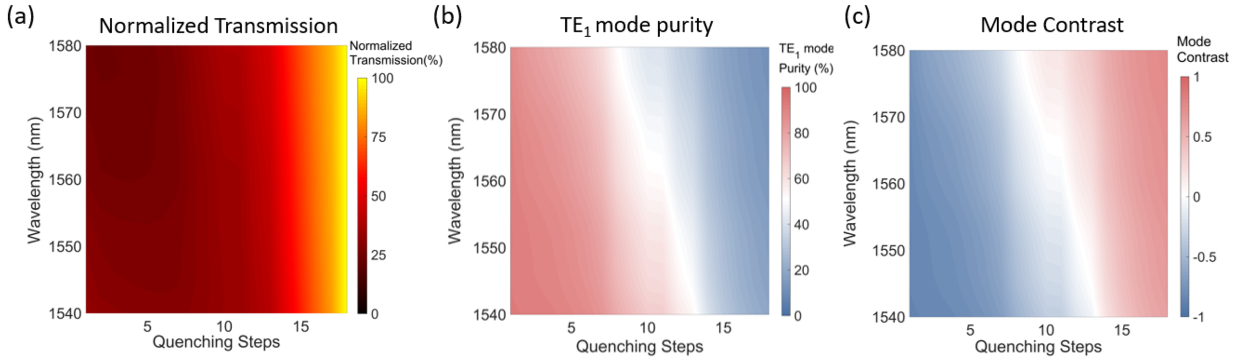

Supplementary Figure 11 Experimental results of the mode converter, showing **(a)** the normalized transmission, **(b)** the TE_1 mode purity, and **(c)** the mode contrast, when both the optical wavelength and the quenching steps are scanned.

Supplementary Note 7: 6-bits nonvolatile programmable mode converter

The phase-change GST has a drastic change in both of its refractive index n and extinction coefficient κ upon phase-transition. The PMMC utilizes the n contrast rather than only κ change between the crystalline and amorphous phases of GST. Thus, the PMMC is much more energy-efficient and performs larger contrast compared to other phase-change material based programmable photonic devices using a similar volume of the GST[7,8]. Ideally, the phase of the GST can be set continuously to an arbitrary level if the controlling pulse sequence is precisely and carefully designed. The number of the output levels will only be limited by the achievable measurement signal-to-noise ratio. The noise level in our experiment is within the range of 0.5% and mainly comes from the noise of photodetectors, and the mechanical vibration between the grating coupler and the fibers. Other than the noise, a larger switching contrast leads to a larger range of change in the output signal with more resolvable levels of change and thus higher programming resolution.

Supplementary Figure 12b shows the programming operation of the PMMC during quench the GST to achieve 88 distinguishable levels in the mode purity. We start with the fully crystalline phase of the GST and progressively quenching GST metasurface toward the aGST phase until the whole metasurface becomes fully amorphous. Each step is achieved by sending into the waveguide a 50 ns duration laser pulse with a gradually increase pulse energy from 43 pJ to 381 pJ. A 500 ns duration pulse with lower pulse energy instead can reset the GST to the fully crystalline phase so the device can be re-programmed. Since the nano-antenna array is offset from the center axis of the waveguide, we use controlling pulses in the TE_1

mode rather than in TE_0 mode to achieve sufficient coupling and thus heating of the GST. Besides high-resolution programmability, the subwavelength patterning the GST nano-antennas, as well as the conformal encapsulation with the Al_2O_3 protection layer, also significantly facilitates achieving the complete phase transition, prevents film deformation and improves the endurance[9]. Supplementary Figure 12a shows five operation cycles, demonstrating that the setting and resetting operations are repeatable and stable.

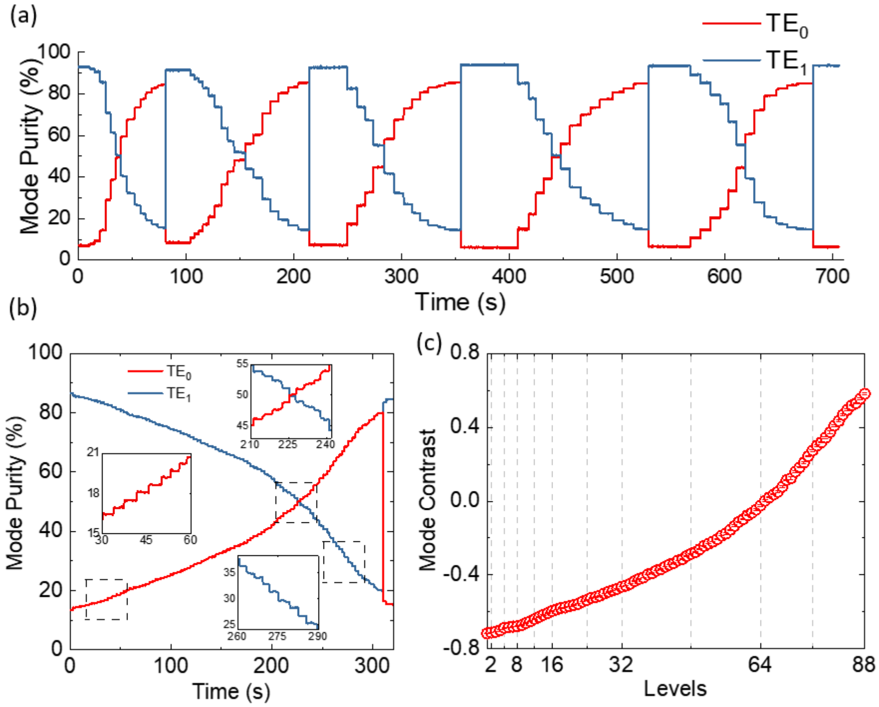

Supplementary Figure 12 Demonstration of programming the PMMC. **(a)** Five programming cycles of setting and resetting the PMMC, showing the PMMC has an excellent repeatability. **(b)** A total of 88 levels in mode purity, which correspond to more than 6 bits of the resolution, can be programmed in the PMMC. The insets show the enlarged details of the plot. Each level can be distinguished clearly. **(c)** The corresponding mode contrast calculated from the mode purity data in Supplementary Figure 12b.

Supplementary Note 8: Repeatability and Endurance Test

We perform testing on the devices' programming repeatability and endurance. The number of programming cycles the device can endure can be affected by two practical factors: 1) the atomic diffusion in the material, and 2) The reflowing, deforming and even ablation of the GST film when the photonic device is programmed. For 30 nm thick GST film we used in this work, the previous estimated up limit due to the material atomic diffusion is larger than 10^5 cycles[11]. We previously also demonstrated that after sub-wavelength patterning and conformal encapsulating the GST with a capping layer (ALD alumina), reflowing and deforming of GST film could be avoided[9].

To demonstrate the endurance of our device, we performed 1000 set/reset cycles of programming of our devices between two arbitrary levels: 1 and 12. The measured mode contrast is plotted in Supplementary

Figure 13b, which shows no evidence of degradation. The slow drift of <2% is mainly due to the drift of our measurement instruments, such as the alignment between the fiber array and the chip. The stability of the device could be future confirmed by comparing the 64 levels of the mode contrast before and after the 1000 cycles. The demonstrated number of cycles is already 10 times more than previously reported in similar photonic devices[7,12]. All the 64 levels are repeated with negligible changes after 1000 cycles. The standard deviation (SD) throughout all 64 levels is only 0.0042.

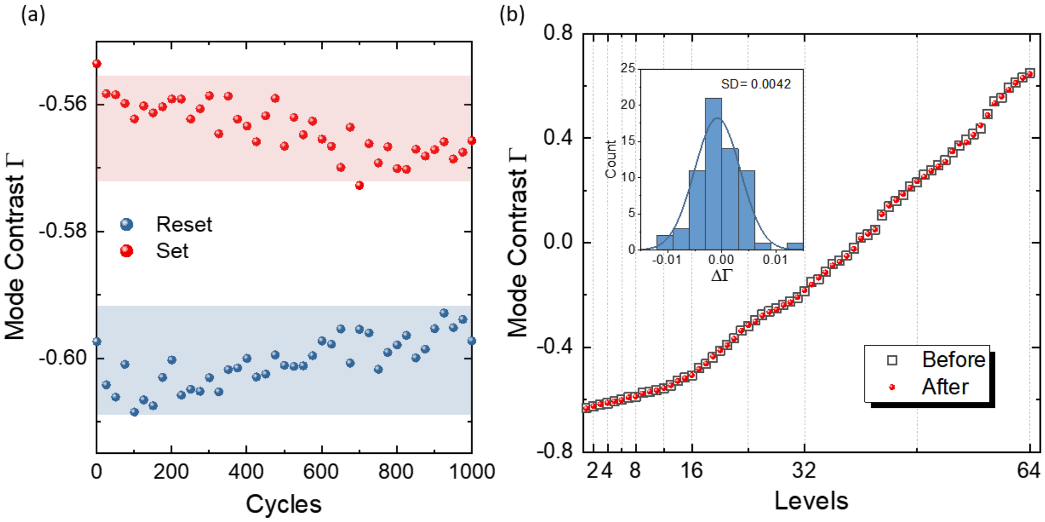

Supplementary Figure 13 Programming repeatability and endurance test. **(a)** 1000 set/reset programming cycles of the PMMC between level 1 and 12, showing only a drift <2% in mode contrast. (b) The 64 levels of mode contrast before and after the 1000 cycles. The standard deviation of variation in contrast throughout all 64 levels is only 0.0042.

Supplementary Note 9: Optical convolutional neural network using PMMCs

1. Encoding 8-bit grayscale image in the optical signal

The first step to perform imaging processing tasks such as edge detection and pattern recognition is to encode the image from 8-bit grayscale into the input optical signal. The 8-bit grayscale data for each pixel, represented by a decimal number between 0 and 255, is first normalized to a value in the range of [0,1]. Experimentally, this value is represented by the transmission coefficient of an optical pulse controlled by an electrical variable optical attenuator (EVOA), with no transmitted power denoting "0" (black) and maximum transmitted power denoting "1" (white). The pixel data of the image encoded in such a way in sent into the PMMCs network in a time sequence of optical pulses. Supplementary Figure 14a shows the calibration result of the EVOA, which controls the attenuation (or transmission) of the laser pulses with an analog voltage between 0 and 5V. To test the stability and accuracy of this process, we generated 10000 random greyscale numbers with a computer and encoded them in the above process. We measured the

encoded optical pulses and compared them with the expected result, as shown in Supplementary Figure 14b. The EVOA is operated at 1 kHz. The measured result is accurate and stable compare to the expected value, and the standard deviation we calculated from the histogram is only 6×10^{-4}. Supplementary Figure 14c and d show the original image we chose to process and the image we recover from the encode optical signal we sent into our network. One can hardly see any difference between these two images, demonstrating that the optical encoding process of a greyscale image is of high fidelity.

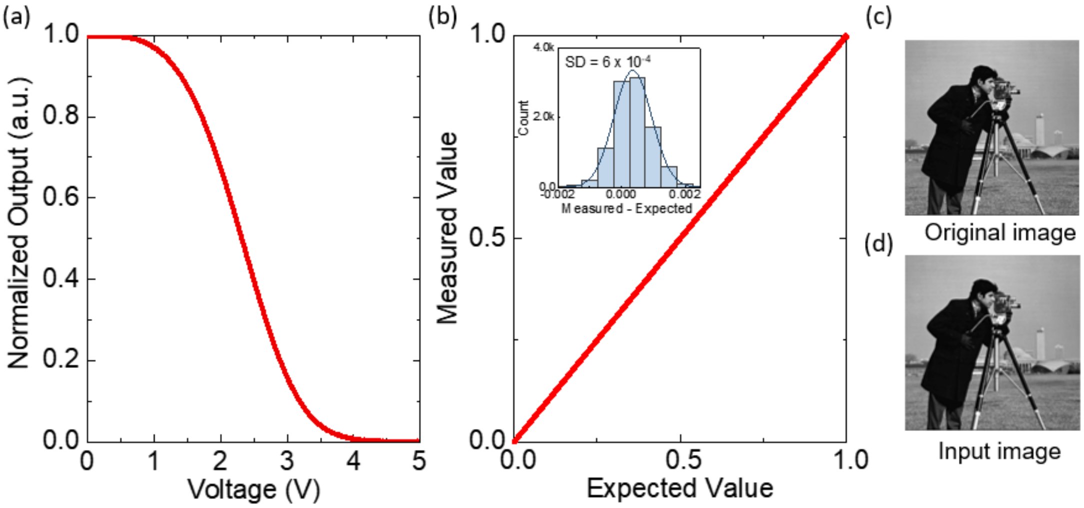

Supplementary Figure 14 (a) Calibration of the normalized transmission of the EVOA as a function of the input voltage. **(b)** 10000 randomly generated integer numbers in [0..255] are normalized and input as a voltage [0-5] V to the EVOA. The EVOA output is measured and plotted after normalization versus the input value. The inset shows the histogram. The standard deviation is only 6×10^{-4}. **(c)** and **(d)**: The original image (c) is encoded in optical signals and sent into the photonic network. The image is recovered from the transmitted optical signal through the network and replotted in (d), which shows no apparent deviation from the original image.

2. Programming the PMMC matrix elements

The next step to perform optical computing with the PMMC array is to store kernel matrices in it. A schematic of our set up for programming the PMMCs is shown in Supplementary Figure 15c. By choosing the corresponding control and probe ports, we selectively program each PMMC individually with the corresponding matrix element. The transmitted TE_1 and TE_0 mode power is measured using two photodetectors to confirm the programmed value.

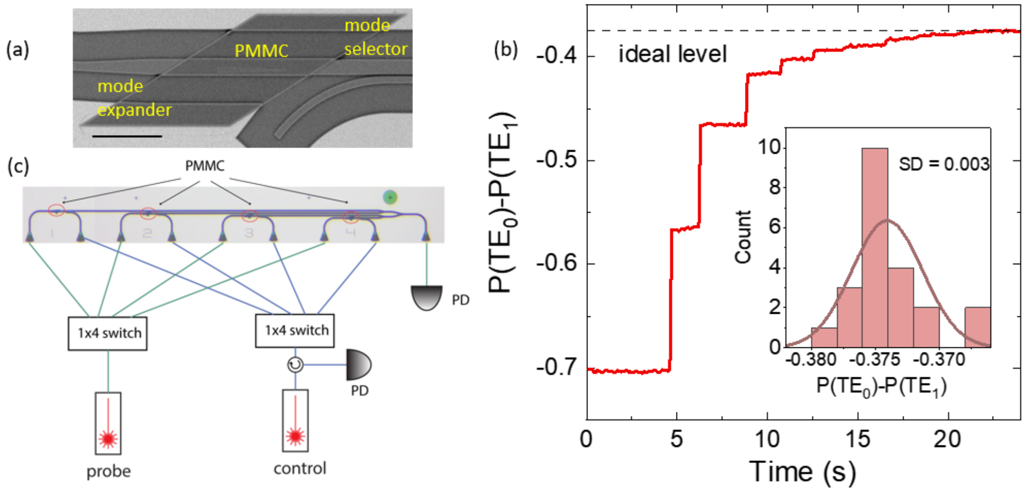

Supplementary Figure 15 (a) SEM image of a PMMC device. **(b)** One typically programming process to set the mode contrast Γ to value -0.375. Inset: histogram of programming error calculated from repeatedly setting the kernel element to the desired value. N=22. The standard deviation is 0.003. **(c)** Schematics of the setup used to program the 2×2 kernel elements.

The mode contrast $\Gamma = (P_{TE0} - P_{TE1}) / (P_{TE0} + P_{TE1})$, is used as the programming parameter. Supplementary Figure 15b demonstrates how we set the kernel element. To set Γ to the desired level (-0.6 in this demonstration), instead of programming with 6-bit precision all the way, we first program “coarsely” using optical pulses with high energy to quickly approach the set level. We then program “finely” using optical pulses with smaller energy. Benefit from the stability and the high precision of the PMMC, the contrast will be set to the desired value precisely, as demonstrated in Supplementary Figure 15b. Besides, the PMMC is re-programmable by resetting it using a 500 ns long reset pulse. We here set Γ to the desired value (-0.375) for 22 times continuously, the programming errors are plotted as a histogram shown in the inset. The standard deviation for our setting is only 0.003, indicating our setting process is accurate and repeatable.

Without loss of generality, we describe the kernel setting procedure as following: first, we determined the input optical power level that represents “white” pixels. Second, we calibrate all PMMCs and determine a scaling factor for each PMMC that compensates for the fabrication fluctuations and fiber alignment variations to equalize their output. This scaling factor is characteristic of each PMMC and, once calibrated, is never changed and used in all the following measurements and operations. Third, we set the kernel matrices to their ideal value of Γ after equalization as the programming parameters. The third step is repeated if the kernel matrices need to be reprogrammed.

3. Edge detection demonstration

The perform the edge detection algorithm, we use the PMMC array photonic core to compute a discrete first-order derivative between adjacent pixels. This corresponds to a convolution operation with the kernel matrix of $\begin{bmatrix} 1 & 1 \\ -1 & -1 \end{bmatrix}$ to highlighting the horizontal edges and $\begin{bmatrix} -1 & 1 \\ -1 & 1 \end{bmatrix}$ for highlighting the vertical edges.

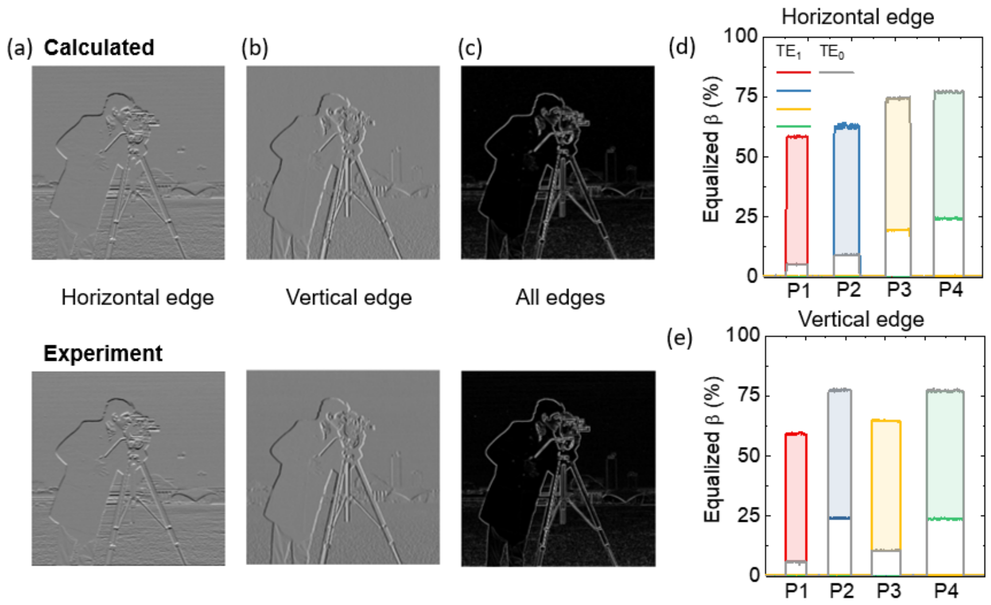

Supplementary Figure 16 (a)-(c): The output images of edge detection, which highlights horizontal (a), vertical (b) and all edges (c). The image processing is achieved by computer calculation (top panel) and photonic CNN (bottom panel), respectively. **(d) (e)**: The equalized TE_0 mode and TE_1 mode purities. Their differences, i.e. mode contrast, correspond to the 2×2 convolutional kernel.

(a) **Horizontal edge**

$$K_x\,(\text{Ideal}) = \begin{bmatrix} -1 & -1 \\ +1 & +1 \end{bmatrix} = \begin{bmatrix} P1 & P2 \\ P3 & P4 \end{bmatrix}$$

	Equalized β_{TE0} (%)	Equalized β_{TE1} (%)	Equalized Γ	Rescale
P1	5.21	57.25	-0.520	-0.984
P2	9.15	61.72	-0.526	-1
P3	72.43	19.15	0.533	1
P4	75.94	24.06	0.519	0.963

(b) **Vertical edge**

$$K_y\,(\text{Ideal}) = \begin{bmatrix} -1 & +1 \\ -1 & +1 \end{bmatrix} = \begin{bmatrix} P1 & P2 \\ P3 & P4 \end{bmatrix}$$

	Equalized β_{TE0} (%)	Equalized β_{TE1} (%)	Equalized Γ	Rescale
P1	5.89	58.18	-0.520	-0.989
P2	76.17	23.75	-0.526	0.993
P3	10.57	63.92	0.533	-1
P4	76.34	23.66	0.519	1

Supplementary Table 2 The ideal value and experimental value of each element of the kernel matrix used to detect the (a) horizontal edge and (b) vertical edge.

We follow the procedure described in Supplementary Note 9.2 and set the kernel matrix. Once the scaling factors are calibrated and the trained kernel matrix elements are programmed, the input 256×256 8 bits grayscale cameraman image is then reorganized into patches and sent into the kernel in the time

sequence. We calculate the output voltage difference between the photodetectors at each time frame and normalize the kernel matrix element from [-0.7,0.7] to [-1,1]. The result is also a series of positive and negative numbers in time sequence, which can be recovered to a 255×255 image. This image highlights the silhouettes of the objects with sharp edges while suppresses smooth backgrounds. Supplementary Figure 16 a,b and c show the images after convolution processing to detect the horizontal, vertical and edges along with both directions. We compare the output images measured using the photonic convolution approach with computer calculated ones, which show an excellent agreement.

4. Training the convolutional neural network to recognize number images

We build and train the convolutional neural network with a standard back-propagation algorithm using the gradient descent method. The network architecture consists of an input layer, a convolutional layer, an average pooling layer, a fully connected layer, and an output layer. The training code is built based on an open-access MATLAB package[10]. When training the network, the learning rate is 0.01, the cost function is the mean square cost function. The training set consists of 11000 images of the handwritten number "1" or "2" from the MNIST database. The epoch number for our training is 200, and the batch size is 20. The training determines the values of all matrices element and bias as shown in Supplementary Figure 17a.

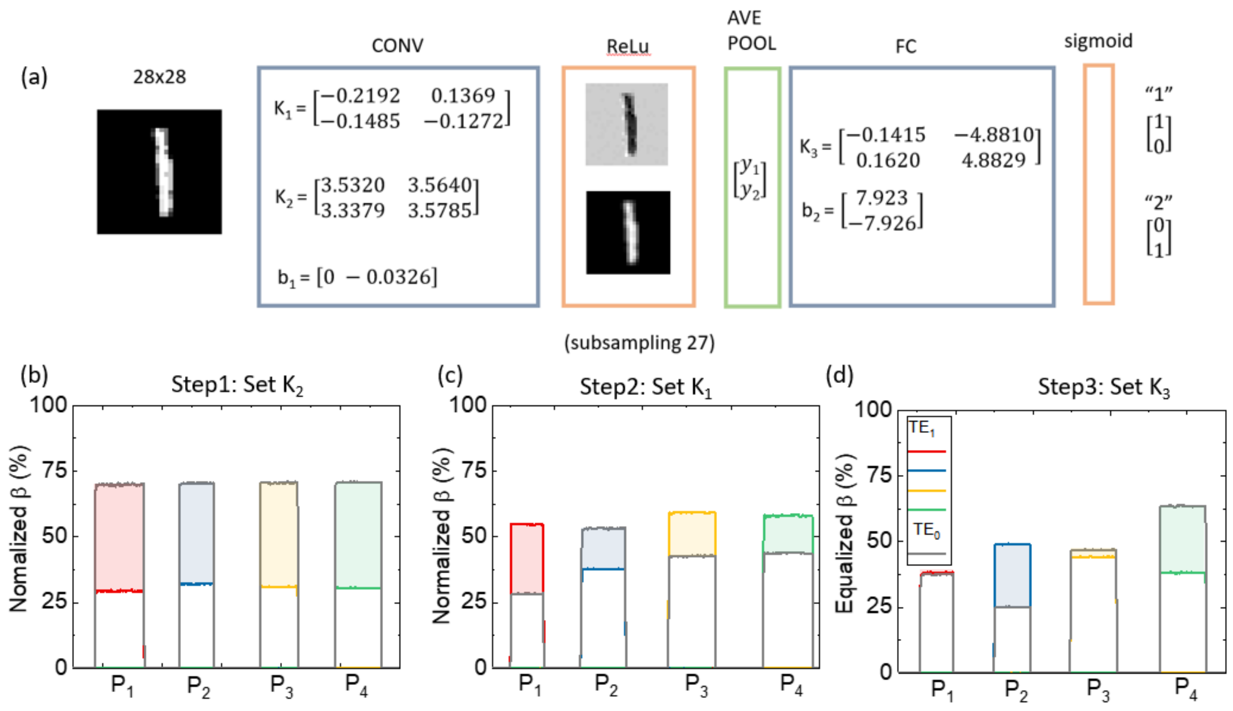

Supplementary Figure 17 (a) Schematic of the CNN configuration showing all the bias and matrices values after training. **(b)-(d)**: The equalized TE_0 mode and TE_1 mode purity of 4 PMMCs respectively. Each element in the 2×2 convolutional kernel $\mathbf{K_2}$, $\mathbf{K_3}$, and the 2×2 weight bank $\mathbf{K_3}$ is encoded in a PMMC as the equalized mode contrast.

Experimentally, we sequentially reuse the PMMC array in both convolution and fully connected layers to demonstrate the corresponding OCNN. We emphasize here that after the network is calibrated, only the kernel matrix can be programmed and reprogrammed while other parameters such as the gain factors of the photodetectors are fixed. We set the kernel matrix following the procedure described in Supplementary Note 9.2. We start by setting the $\mathbf{K_2}$ then $\mathbf{K_1}$ matrix. A hundred randomly chosen handwritten images of "1" and "2" are encoded as the input signal and convolves with the kernel matrices. After the convolution process, post-processing is performed to the output electrical signal to add bias and apply nonlinear function and pooling. The resultant output is reorganized as 100 2×1 vectors in total and multiplied with the 2×2 weight bank $\mathbf{K_3}$, which is programmed in the PMMC array. In some of the processes described above, rescaling is performed to equalize the transmission difference of different channels. The final result is 100 2×1 vectors, with the first element gives the score for the class "1" while the second element gives the score for the class "2". If the first element is larger than the second element, we identify the image as number "1", otherwise, we identified it as "2". We test 100 randomly chosen "1" or "2" images from the MNIST testing image database Our OCNN correctly identified 91 out of 100 cases (9% error rate), which is consistent with the result of a computer (10% error rate). The slight difference is mainly caused by the small deviation of the experimentally programmed values from the ideal ones due to the system's conditions drift during operation.

(a) Step 1 Setting K_2 (Ideal) = $\begin{bmatrix} 3.5320 & 3.5640 \\ 3.3379 & 3.5785 \end{bmatrix}$

	Equalized β_{TE0} (%)	Equalized β_{TE1} (%)	Equalized Γ	Rescale
P1	68.98	28.98	0.400	3.60
P2	69.13	31.50	0.376	3.38
P3	69.44	30.56	0.389	3.50
P4	69.83	30.09	0.397	3.57

(b) Step 2 Setting K_1 (Ideal) = $\begin{bmatrix} -0.2192 & 0.1369 \\ -0.1485 & -0.1272 \end{bmatrix}$

	Equalized β_{TE0} (%)	Equalized β_{TE1} (%)	Equalized Γ	Rescale
P1	27.55	53.36	-0.258	-0.231
P2	52.13	37.05	0.151	0.135
P3	41.90	58.08	-0.162	-0.145
P4	43.02	56.98	-0.140	-0.125

(c) Step 3 Setting K_3 (Ideal) = $\begin{bmatrix} -0.1415 & -4.8810 \\ 0.1620 & 4.8829 \end{bmatrix}$

	Equalized β_{TE0} (%)	Equalized β_{TE1} (%)	Equalized Γ	Rescale
P1	32.88	33.46	-0.006	-0.122
P2	24.53	47.90	-0.234	4.739
P3	46.05	43.45	0.026	0.526
P4	62.47	37.53	0.249	5.04

Supplementary Table 3 The ideal value and experimental value of each element of the kernel matrices $\mathbf{K_1}$, $\mathbf{K_2}$ and weight bank $\mathbf{K_3}$ in the OCNN.

Supplementary Note 10: The perspective of scalability— crossbar array architecture

1. Work principle of the crossbar array

We have demonstrated an OCNN and perform proof-of-concept imaging recognition tasks of distinguishing handwritten numbers "1" and "2" from the MNIST database using the prototype device (See Fig. 3 of the main text or Supplementary Figure 7). The network architecture is shown in Fig. 3 of the main text can be scaled up using a crossbar array architecture as shown in Supplementary Figure 18.

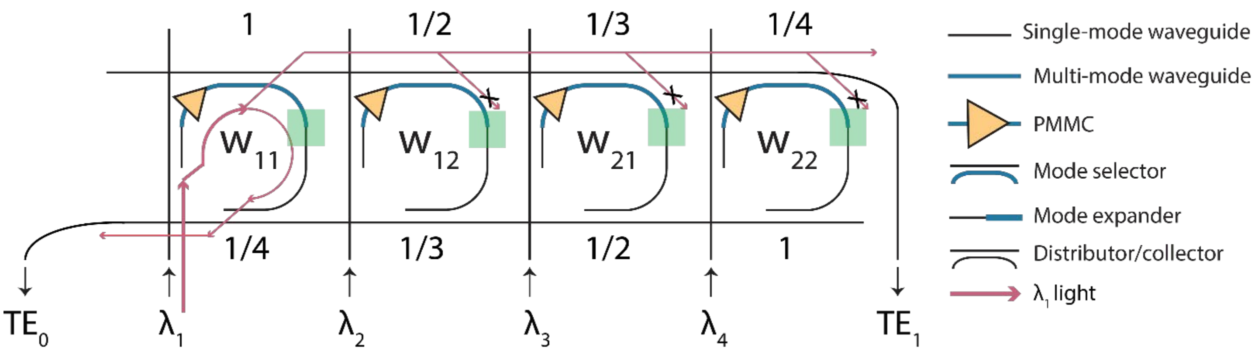

Supplementary Figure 18 (a) Schematic of a photonic crossbar array architecture used to perform optical convolution operation with a 2×2 kernel matrix. The arrows denote the detailed optical path for the first input channel (λ_1). The cross-coupling ratios for each mode selector and mode expander are labeled, respectively.

All the TE_1 output from each of the 4 units are combined incoherently in the top horizontal waveguide and summed at the output. Likewise, all the TE_0 output are combined in the lower horizontal waveguide and summed. It is thus important that the output from each unit is combined with the same weight. This is achieved by the arrangement of the directional couplers between each unit and the bus waveguide. We elaborate the steps below:

The input TE_0 mode in the first channel with wavelength λ_1 passes through the 1st unit of PMMC, which corresponds to the kernel element w_{11}, and is partially converted to TE_1 mode based on the value of w_{11}. So this is doing the part of the multiplication of $x_1 \cdot w_{11}$ (after taking a difference from the TE_0 mode). The TE_1 mode component couples to the top bus waveguide with a 100% coupling ratio and then passes by the next three units before reaching the output. In the 2nd unit with kernel element w_{12}, the coupling ratio between the PMMC and the bus waveguide needs to be designed to be 1/2, so the through port efficiency of the bus waveguide is also 1/2. For the 3rd unit with kernel element w_{13}, the PMMC to bus coupling efficiency should be 1/3 and the through port efficiency of the bus is 2/3. Finally, in the 4th unit, the two coupling ratio should be 1/4 and 3/4, respectively. Therefore, the overall collective efficiency of the TE_1 mode output from the 1st unit will be $1 \times 1/2 \times 2/3 \times 3/4 = 1/4$. Similarly, the overall collective efficiency of the TE_1 mode output

from the 2nd unit will be 1/2 × 2/3 × 3/4 = 1/4. For the 3rd and 4th units, the overall efficiency will be the same. The collective efficiencies for the TE_0 mode power from each unit to the bottom bus waveguide are designed in the same way to be 1/4. These coupling efficiencies are denoted in the figure above. Also note that the TE_1 light left in multimode waveguide will be filtered out by the mode expander (the green box) so will not be collected by the lower bus waveguide. More details about the mode expander performance can be found in Supplementary Note 5.

2. Mapping convolutions to photonic MAC operations

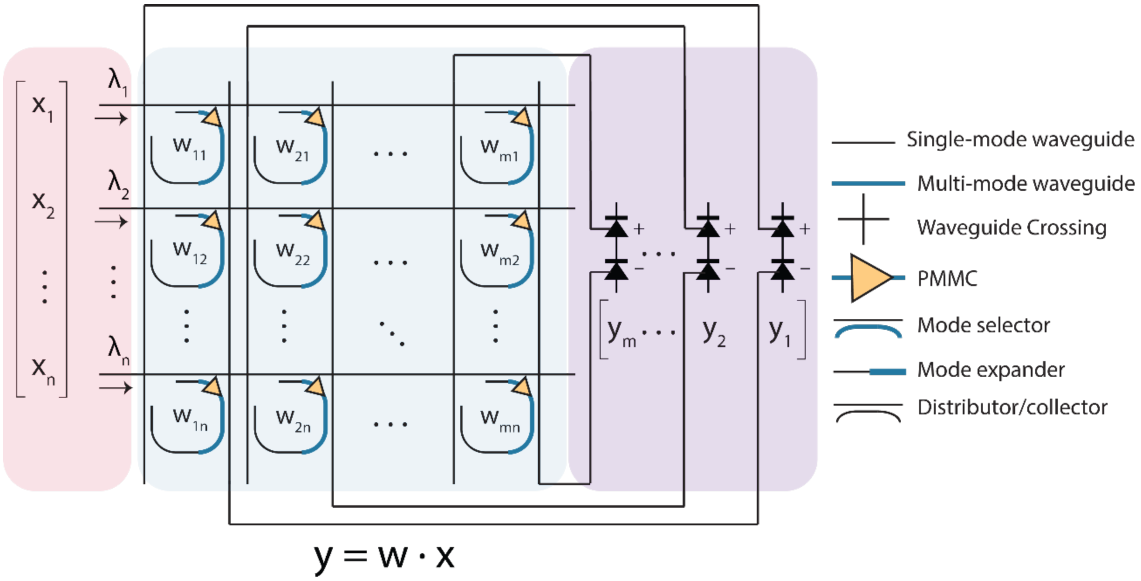

Supplementary Figure 19 Schematic of a large photonic crossbar array used to perform MVM operation. The horizontal and vertical waveguides separate the network into $m \times n$ subunits.

To further scale up our system to realize large convolution kernels in parallel, a photonic crossbar array architecture as sketched in Supplementary Figure 19 can be used. The photonic crossbar array can perform a general MVM operation: $\mathbf{y}=\mathbf{w}\cdot\mathbf{x}$, where $\mathbf{x}=[x_1, x_2, \ldots, x_n]^T$ is the input vector, the $\mathbf{w}$ is the $m \times n$ matrix represents m convolutional kernel matrices in parallel $\mathbf{y}=[y_1, y_2, \ldots, y_m]^T$ is the output vector. The horizontal and vertical single-mode waveguide separates the whole structure into $m \times n$ subunits. The input vector $\mathbf{x}$ is encoded through a group of wavelength $[\lambda_1, \lambda_2, \ldots, \lambda_n]$, with the value of each element x_i represented by the input power, and sent into n horizontal bus waveguides. Directional coupler based power distributor is used to distribute the power carried by bus waveguide evenly into m subunits. These m subunits in a row of the crossbar array correspond to a column in the matrix $\mathbf{w}$. The $2m$ vertical single-mode waveguides are used as power collectors to collect the light after leaving each PMMC. The TE_0 and TE_1 lights collected and summed from the same row in the matrix w (the same column in the crossbar array) will be grouped in pairs

and measured with balanced photodetectors to determine the contrast. Both the photonic convolution and the MVM operations are based on the photonic MAC operations, the *m* convolution kernels with the dimensions of $\sqrt{n} \times \sqrt{n}$ thus can be mapped to the *w* matrix. The operation volume can be further extended through the wavelength-division multiplexing (WDM) method. For example, if *g* groups of the wavelength are implemented parallelly into the crossbar network with *n* wavelength per group, the numbers of the MAC operation performed can increase by *g* times, $g \times m \times$ n MAC operations or $g \times m$ convolutions can be applied in parallel.

3. The limiting factors of scalability and comparison with the state-of-the-art commercial microprocessors

The insertion loss that is for the TE_1 mode when the GST is in cGST phase (TE_1 mode at aGST phase and TE_0 mode at cGST mode is supposed to be suppressed). As shown in Fig. 2d in the main text, this insertion loss (green triangle symbols) of our PMMC device is ~7 dB in the measured wavelength range. Although lower this insertion loss is beneficial to scaling, it is not the bottleneck for a crossbar array architecture that is commonly used. This is because the optical signal in each channel only passes one PMMC once to perform the multiplication: $w_{ij} \cdot x_i$. Rather, in a large network, the optical power loss will be dominated by the directional couplers. To equalize the output from *n* units, the directional couplers that are used to collect the output from each unit to the bus waveguide need to be designed to achieve a 1/*n* weight for each unit. Therefore, when $n>5$, this loss will be larger than the 7 dB insertion loss of the PMMC.

With these numbers, we can estimate what is a crossbar array size limited by the optical loss, assuming no optical amplifiers are used to boost up the power. If we assume the input optical power for a single wavelength channel is at a moderate level of 10 mW (10 dBm), and a 10 GHz bandwidth photoreceiver with noise-equivalent power of 10 $pW/\sqrt{Hz}$ (e.g. RXM10AF from Thorlabs) is used. So 40 dB total insertion loss is allowed. This means *n* can be as large as 2000, considering 7 dB loss of a PMMC (40-7 dB=33 dB, approximately 1/2000). This is already a very large network, corresponding to a kernel matrix of ~45×45, or 45 5×9 kernel matrices in parallel. To go beyond this size limitation imposed by device insertion loss, optical amplifiers will need to be inserted in the network. It is possible to integrate semiconductor optical amplifiers (SOA) in the photonic network. The amplifiers, however, will increase the noise figure and negatively impact the accuracy of the network.

The photonic crossbar array architecture with WDM can fully utilize the intrinsic parallelism of photonic systems. We can estimate and compare the expected computation performance, including speed (Tera-Operations per second (TOPS)), operation power, clocks, MAC sizes, computing density, energy efficiency (TOPs/W), of the PNN with commercial GPUs commonly used in as accelerators for neural network-based AI applications.

We assume an array size of 32×32, an operation speed of 10 Gbits/sec in data rate, and 16 wavelengths used in WDM. All of these values are quite moderate compared with the state-of-the-art optical communication technology. Since our PMMC device is very compact, its footprint (including the mode selector) is only 80×20 μm^2, a 32×32 array will an area of less than 2 mm^2. With 10 Gbit/sec operation frequency and 16 wavelengths, the network's computation speed will be 10G/sec×16×32×32=164 TOPS. Its areal computing density will thus be 82 TOPS/mm^2. As shown in the Supplementary Table 4, these values compare very favorably with the current digital computing technology for neural networks, such as GPUs and TPUs. In terms of computation density (TOPS/mm^2), the photonic architecture is 800× higher than GPUs, 60× higher than TPUs, and 20× higher than the emerging memristor processors.

Processor	Format	TOPS	Clock [GHz]	MAC Size	MAC Area (estimated) [mm^2]	TOPS/mm^2
Nvidia GPU P40	INT 8	48	1.3GHz	-	471	0.1
Nvidia GPU V100	INT 8	62.8	1.3GHz	-	815	0.08
Google TPU	INT 8	90	700MHz	256×256	72	1.25
IBM (Memristor)	INT 8	1.4	4.2MHz	512×512	0.4	3.6
**Photonic CNN (16-λ)**	**INT 6**	**164**	**10GHz**	**32×32**	**2**	**82**

Supplementary Table 4 Comparison of the projected performance parameters of a photonic CNN with the commercial electronic processors.

Reference:

1. Chen, Y., Li, X., Sonnefraud, Y., Fernández-Domínguez, A.I., Luo, X., Hong, M. and Maier, S.A., Engineering the phase front of light with phase-change material based planar lenses. *Scientific reports*, 2015, **5**(1), 1-7.
2. Voshchinnikov, N.V., Videen, G. and Henning, T., Effective medium theories for irregular fluffy structures: aggregation of small particles. *Applied Optics*, 2007, **46**(19), 4065-4072.
3. Chakraborty, I., Saha, G. & Roy, K. Photonic In-Memory Computing Primitive for Spiking Neural Networks Using Phase-Change Materials. *Physical Review Applied* 11, 014063, doi:10.1103/PhysRevApplied.**11**.014063 (2019).
4. Li, Z. et al. Controlling propagation and coupling of waveguide modes using phase-gradient metasurfaces. *Nat. Nanotechnol.* 12, 675 (2017).
5. Zhang, Y., Chou, J.B., Li, J., Li, H., Du, Q., Yadav, A., Zhou, S., Shalaginov, M.Y., Fang, Z., Zhong, H. and Roberts, C., Broadband transparent optical phase change materials for high-performance nonvolatile photonics. *Nature communications*, 2019, **10** (1),1-9.
6. Q. Zhang, Y. Zhang, J. Li, R. Soref, T. Gu, and J. Hu, "Broadband Non-volatile Photonic Switching Based on Optical Phase Change Materials: Beyond the Classical Figure-of-Merit," Opt. Lett. 43, 94-97 (2018).
7. Zhang, H. et al. Miniature Multilevel Optical Memristive Switch Using Phase Change Material. ACS Photonics 6, 2205-2212, doi:10.1021/acsphotonics.9b00819 (2019).
8. Li, X. et al. Fast and reliable storage using a 5-bit, nonvolatile photonic memory cell. Optica 6, 1-6 (2019).
9. Wu, C. et al. Low-Loss Integrated Photonic Switch Using Subwavelength Patterned Phase Change Material. ACS Photonics 6, 87-92, doi:10.1021/acsphotonics.8b01516 (2018).
10. Convolution Neural Network - simple code - simple to use (MATLAB Central File Exchange, 2020).
11. Simpson, R. E., et al. "Interfacial phase-change memory", Nature Nanotechnology 6, 501 (2011).
12. Cheng, Z., et al. Device-Level Photonic Memories and Logic Applications Using Phase-Change Materials. Advanced Materials, 30, 1802435 (2018).
